# Development of Response Surface Model of Endurance Time and Structural Parameter Optimization for a Tailsitter UAV

**DOI:** 10.3390/s20061766

**Published:** 2020-03-22

**Authors:** Xiaomin Yao, Wenshuai Liu, Wenting Han, Guang Li, Qian Ma

**Affiliations:** 1 College of Mechanical and Electronic Engineering, Northwest A & F University, Yangling 712100, China; yaoxiaomin0604@163.com (X.Y.); liuwenshuai@nwafu.edu.cn (W.L.); liguang@nwafu.edu.cn (G.L.); 2 Institute of Soil and Water Conservation, Northwest A & F University, Yangling 712100, China; 3 Institute of Soil and Water Conservation, CAS&MWR, Yangling 712100, China; MQ2020@126.com

**Keywords:** tailsitter, structural optimization, MOGA, RSM

## Abstract

This study designed a vertical take-off and landing tailsitter unmanned aerial vehicle (UAV) with a long endurance time. Nine parameters of the tailsitter UAV were investigated. Using a 2^k^ full factorial test, 512 experiments on the nine parameters were conducted at their maximum and minimum values. The time coefficient and air resistance were calculated using the computational fluid dynamics (CFD) method under different parameter combinations. The analysis of variance determined that the specific factors influencing the time coefficient and air resistance were the root chord, wingtip chord, wingspan, and sweep angle. By carrying out a central composite design (CCD) test, 25 sample points of the four particular factors were constructed. The time coefficient and air resistance were simulated under different structural parameter combinations using the CFD method. CFD simulation was verified by carrying out a wind tunnel test, and the results revealed that the aerodynamic coefficient error was less than 5%, while the air resistance error was less than 6%. The response surface methodology (RSM) for the time coefficient and air resistance was established using a genetic aggregation method. A multi-objective genetic algorithm (MOGA) was used to optimize the parameters with regard to the maximum time coefficient and minimum air resistance. The optimal structural parameters were wing root chord length at 315 mm, wingtip chord length at 182 mm, wingspan length at 1198 mm, and sweep angle at 16°. Compared with the original layout and size, the time coefficient of the new design of the tailsitter UAV improved by 19.5%, while the air resistance reduced by 34.78%. The results obtained by this study are significant for the design of tailsitter UAVs.

## 1. Introduction

The tailsitter unmanned aerial vehicle (UAV) has multi-rotor vertical take-off and landing (VTOL) characteristics and fixed-wing dynamic cruising characteristics. These characteristics solve the low efficiency and short-range of failures in multi-rotor UAVs, the lengthy fixed-wing UAV placement preparation, auxiliary equipment requirement, and various landing problems. Additionally, tailsitter UAVs can satisfy the requirements of increasingly complex agricultural low-altitude remote sensing tasks [1,2,3,4,5]. Compared with other types of VTOL vehicles, the tailsitter UAV does not need additional rotating control units and has the advantages of a compact structure, a light weight, high overall natural stability, and smooth operation.

Many studies used computational fluid dynamics (CFD) in tailsitter UAV design and aerodynamic optimization [6,7,8,9,10,11]. In 2014, Ang et al. designed a foldable drone U-lion using a CFD numerical simulation to compare and analyze the lift–drag ratio and polar curve of different wings and completed the structural design and parameter optimization of the wings [12]. In 2017, Wang et al. designed a four-rotor drone and analyzed the changes in the lift coefficient, torque, and polar curve of the drone with different angles of attack from 0° to 90° [13]. In 2012, Rao et al. conducted CFD simulations to analyze the aerodynamic coefficient changes of UAVs with different angles of attack, developed a six degrees of freedom dynamic model, and performed a flight data analysis for the UAVs [14]. Yun et al. evaluated the power system’s performance by carrying out numerical simulation analyses for the lift efficiency of the lift fan system in hover mode, lift and drag in transition mode, airplane resistance in cruising mode, and aerodynamic coupling of the blade tip turbine and jet engine [15]. Cetinsoy et al. compared and analyzed the effects of different airfoil shapes and sizes on the aerodynamic characteristics of tilting wing UAVs; and ultimately determined the structural parameters of the airfoils [16]. Czyba et al. used CFD to analyze the lift–drag ratio change in three modes (VTOL, transition conversion, and horizontal flight), and optimized the structural parameters of the wing and propeller installation parameters [17]. Seong et al. analyzed the changes in the flow field of the fuselage with different rotor angles, and the changes in the lift and resistance of the wings. Additionally, the authors analyzed the aerodynamic characteristics of the rotor and fuselage under different operating conditions [18]. Aksugur and Inalhan designed a ducted tailsitter drone and used CFD to carry out a dynamic simulation analysis for the different motion states of the drone [19].

As a commonly used structural optimization tool for aircrafts, CFD can accurately simulate and calculate the aerodynamic characteristics of drones. However, the CFD method can only obtain discrete data points, and its use for determining global optimal structural parameters is challenging. The response surface model and multi-objective genetic algorithms have been widely used as global optimal solutions in the engineering field [20,21]. For example, in 2008, Che and Tang used a multi-objective genetic algorithm to optimize the structural parameters of an aspirated hypersonic cruise vehicle (HCV) with its maximum lift–drag ratio, minimal drag coefficient, and minimum radar scattering cross-section (RCS). The lift–drag ratio of the optimized design increased by 30%, the drag coefficient decreased by 21%, and the RCS decreased by 21% [22]. Hutagalung et al. optimized the wing structure of a composite UAV by considering the tensile strength and bending stiffness as the response targets [23]. Lee et al. used a genetic algorithm and a hybrid genetic algorithm to optimize the design of the leading edge angle, trailing edge angle, and actuator installation position of the deformed wing. The optimized takeoff lift coefficient increased by 44.4%, while the landing lift coefficient increased by 25.7% [24]. Ganguli and Rajagopal used a multi-objective genetic algorithm called the non-dominated sorting genetic algorithm-II (NSGA-II) to optimize the structural parameters of the airfoil and wing, respectively. After optimization, the maximum lift coefficient of the airfoil increased by 13%, the maximum durability increased by 44%, and the wing weight decreased by 36% [25].

Genetic algorithms provide an alternative to traditional optimization techniques by using directed random searches to locate optimal solutions in complex landscapes. In 2005, He and Zhang integrated genetic algorithms with response surface methodology, as well as designed and optimized the parameters of genetic algorithms to prevent the genetic algorithms from reaching local convergence [26]. Acar introduced this method to structural parameter optimization in 2010 [27]. Zhu et al. used response surface and genetic algorithms to optimize the number of wing layers and the position of the spar installation; the weight was reduced by 9.28% after optimization [28]. Wang et al. used response surface and multi-objective genetic algorithms to optimize the four structural parameters of the heat exchanger’s layer spacing, winding angle, tube spacing, and tube outer diameter. The optimized heat exchanger’s heat transfer coefficient increased by 3%, and the pressure drop decreased by 40% [29].

Endurance time is an essential parameter affecting the general performance of UAVs. However, existing studies have mainly focused on flight parameters and control algorithms. For example, in 2011, Aksugur and İnalhan proposed a new hybrid UAV design concept and carried out an optimized design of a hybrid propulsion system [30]. In 2016, Wang et al. carried out a combined analysis on flight space, wing load, and batteries to optimize the flight parameters of electric-driven tailsitter drones [31]. In 2015, Wang et al. used the battery discharge curve and flight parameters to estimate spatial distance [32]. The structure and layout of a tailsitter drone is an essential factor that affects its battery life. However, existing research cannot explain the relationship between battery life and structural parameters.

With regard to the design of a long endurance tailsitter UAV, this study investigated the relationship between structural parameters and endurance time using a genetic clustering algorithm, and used a multi-objective genetic algorithm to optimize the drone’s structural parameters.

## 2. Materials and Methods

### 2.1. Structural Design and Parameter Range of the Tailsitter UAV

#### 2.1.1. UAV Structure Layout

**Figure 1:**
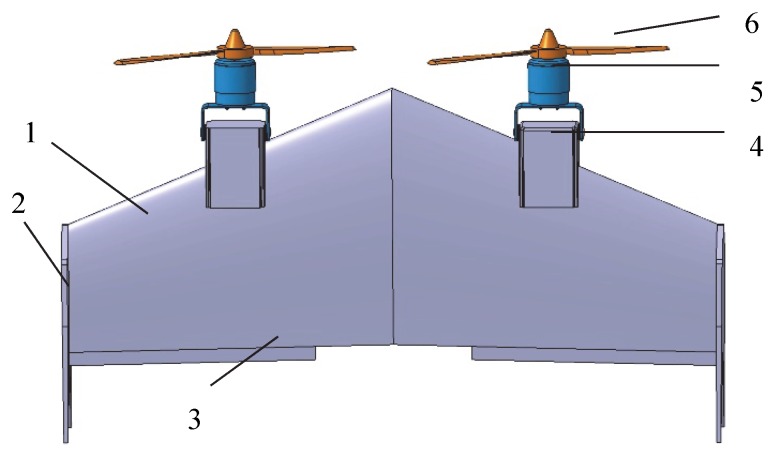
Tailsitter unmanned aerial vehicle (UAV) schematic. (1) wing; (2) winglet; (3) rudder surface; (4) motor seat; (5) motor; (6) propeller.

The tailsitter UAV adopts a double-pull forward layout with flying wings, as shown in Figure 1. It has a left–right symmetrical structure, including the wings, winglets, rudder surface, motor seats, motors, and propellers. When the drone takes off, it performs a vertical ascent in the multi-rotor flying mode, and the rudder surface performs attitude control and adjustment. After reaching a predetermined height, the drone enters into the transition flying mode, the motor speed increases, and the rudder surface provides a lowering torque for transformation into a fixed-wing flying mode. Then, the UAV collects the remote sensing images of the working area while cruising. Before landing, the motor speed decreases to reduce the flying velocity. When it reaches a certain speed, the rudder surface provides a head-up torque to pull the nose. During landing, the drone enters into the multi-rotor flying mode again.

During the whole flight course, the fixed-wing flying mode accounts for more than 80% of the total battery life. In this study, the development of a response surface model of endurance time for the tailsitter drone was mainly during the fixed-wing flying mode.

#### 2.1.2. Structural Parameter Range

**Figure 2:**
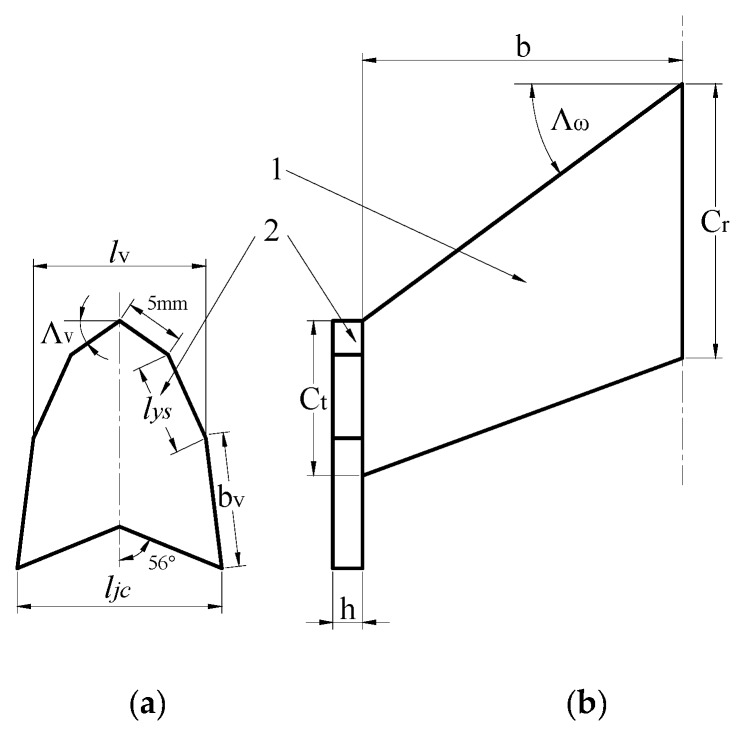
Structural parameters affecting endurance time. (**a**) Winglet structural parameters; (**b**) top view of the UAV. (1) wing; (2) winglet.

The main structural parameters (Figure 2) affecting the drone’s endurance time are the wingspan *b*, root chord *c_r_*, wingtip chord *c_t_*, wing sweep angle *Λ_w_*, winglet length *l_ys_*, winglet span *b_v_*, winglet height *l_v_*, winglet swept angle *Λ_v_*, winglet thickness *h*, and winglet foot length *l_jc_* (the distance between the two legs of the winglet).

Switzerland’s senseFly and senseFly eBee, France’s Parrot disco-pro, and China’s skywalker X5 are all electric driven fixed-wing remote sensing UAVs currently in agriculture. The UAV parameters obtained from the manufacturer’s official website are listed in Table 1.

The range of *b* can be determined by the wingspan range of the existing UAV presented in Table 1. The scope of *c_r_* (240–500 mm) was determined by considering the embedded flight control installation, digital transmission system, and other equipment. The range of *c_t_*, *Λ_w_*, *Λ_v_*, and *l_v_* can be carried out according to the small flying wing drone’s taper ratio (0.6) [33], sweepback angle (0–60°) [34], winglet sweep angle (30–60°), and winglet height (0.05*b*–0.3*b*) range [35,36]. The expanded polypropylene (EPP) material stiffness is considered to determine the *h* range. The range of *b_v_*, *l_ys_*, and *l_jc_* was selected to determine the longitudinal stability of the UAV’s VTOL timing body. The sensitivity analysis results showed that the influences of parameter *b_v_* on air resistance and the time coefficient are very small. The sensitivity coefficient of air resistance is 0.4% and 0.2% the time coefficient. Therefore, parameter *b_v_* was set to a fixed value (180 mm) to reduce computation load. The final structural parameter range is presented in Table 2.

### 2.2. Governing Equations and Numerical Method

The tailsitter UAV combines the advantages of a multi-rotor VTOL and fixed-wing efficient cruising but also has the disadvantages of short endurance time and poor attitude conversion stability. Therefore, improving the aerodynamic efficiency, extending the endurance time, reducing the air resistance, reducing the lateral slip, and enhancing the security of the attitude conversion are key considerations for optimizing the UAV’s structural parameters. Additionally, the CFD numerical simulation method can be used to simulate the external flow field of the UAV.

The equations governing the continuity, momentum, and energy in the computational domain can be expressed as follows:

Continuity:

(1) $$\frac{\partial }{\partial x_{i}}(\rho u_{i})=0.$$

Momentum:

(2) $$\frac{\partial }{\partial x_{i}}(\rho u_{i}u_{k})=\frac{\partial }{\partial x_{i}}(\eta \frac{\partial u_{k}}{\partial x_{i}})-\frac{\partial p}{\partial x_{k}}.$$

Energy:

(3) $$\frac{\partial }{\partial x_{i}}(\rho u_{i}t)=\frac{\partial }{\partial x_{i}}(\frac{\lambda }{c_{p}}\frac{\partial t}{\partial x_{i}}).$$

The *k-ω-*based shear stress transport (SST) model provides highly accurate predictions for the onset and amount of flow separation under adverse pressure gradients by considering the transport of turbulent shear stress.

*k*-equation:

(4) $$\frac{\partial (\rho k)}{\partial t}+\frac{\partial }{\partial x_{j}}(\rho U_{j}k)=\frac{\partial }{\partial x_{j}}\left[\left(\mu +\frac{\mu_{t}}{\sigma_{k}}\right)\frac{\partial k}{\partial x_{j}}\right]+P_{k}-\beta^{\prime }\rho k\omega {+P}_{\mathrm{kb}}$$

*ω*-equation:

(5) $$\frac{\partial (\rho \omega )}{\partial t}+\frac{\partial }{\partial x_{j}}(\rho U_{j}\omega )=\frac{\partial }{\partial x_{j}}\left[\left(\mu +\frac{\mu_{t}}{\sigma_{\omega }}\right)\frac{\partial \omega }{\partial x_{j}}\right]+\alpha \frac{\omega }{k}P_{k}-\beta \rho \omega^{2}{+P}_{\omega b}$$

In addition to the independent variables, the density ρ and velocity vector U are treated as known quantities in the Navier–Stokes method; $P_{k}$ is the production rate of turbulence and is calculated in the same manner as in the k−ε model. The model constants are as follows: $\beta^{\prime }=0.09$, α=5/9, and β=0.075.

The proper transport behavior can be obtained by applying a limiter to the formulation of eddy-viscosity, as follows:

(6) $$v_{t}=\frac{a_{1}k}{\max(a_{1}w,SF_{2})}$$

where

(7) $$v_{t}=\frac{\mu_{t}}{\rho }.$$

Again, $F_{2}$ is a blending function restricting the limiter to the wall boundary layer because the underlying assumptions are not valid for free shear flows; S is an invariant measure of the strain rate.

$$F_{2}=\tanh(\arg_{2}^{2})$$

$$\arg_{2}=\max(\frac{2\sqrt{k}}{\beta^{\prime }\omega y},\frac{500\nu }{y^{2}\omega })$$

**Figure 3:**
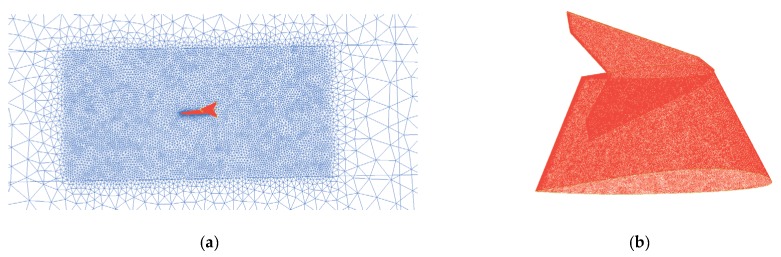
Outflow mesh. (**a**) local mesh refinement; (**b**) UAV surface mesh.

The three-dimensional model of the UAV was built by computer aided tri-dimensional interface application (CATIA), which is widely used in aircraft design [37]. Owing to the symmetrical layout of the UAV, a three-dimensional model was built for the left side of the fuselage, and the structures of the propeller, motor, and motor seat were simplified. The UAV 3D model was imported into the ansys (ANSYS) geometry module to create the outflow field. The outflow field was constructed with the dimensions of 5 × 5 × 3 m, and the three-dimensional solid model of the outflow field was obtained by a Boolean operation [38]. The grid of the outflow field was generated with the ICEM software [39,40]. An unstructured grid was used for grid generation. Additionally, to capture the flow details of the UAV close to the wall and outside the flow field, the grid around the UAV was locally encrypted, as shown in Figure 3a. The number of the final generated grid of the outflow field was 2.3 million nodes, and the grid close to the wall of the UAV is shown in Figure 3b.

Fluent is a common CFD software that is widely used in the optimal design of aircraft and can accurately calculate aerodynamic parameters. The lift coefficient *C_L_*, drag coefficient *C_D_*, and air resistance of the UAV were calculated by Fluent with the external flow field [41,42,43,44].

The boundary conditions in Fluent software were set as follows: the inlet speed was 12 m/s, the included angle was 4–12°, and the outlet pressure was standard atmospheric pressure. The working fluid was air, and its physical properties were considered constant. For the boundary of the calculation area, no-slip conditions and the standard law for the wall were assumed. The semi-implicit method for pressure-linked equations (SIMPLE) method in Fluent software was adopted for the coupling of pressure and velocity. The convergence criterion was considered as normalized residuals being less than 1 × 10^−4^ for the flow equations.

### 2.3. Response Surface Model of Endurance Time

#### 2.3.1. Theoretical Model of Endurance Time

The endurance time is mainly related to the battery power, take-off weight, and aerodynamic characteristics. According to Tan [45], the endurance time can be expressed as follows:

(8) $$t=Q\sqrt{\frac{\rho s}{2G^{3}}}\times \frac{C_{L}{}^{\frac{3}{2}}}{C_{D}}$$

In this study, $G_{FA}=\sqrt{\frac{\rho s}{2G^{3}}}$, $C_{FA}=\frac{C_{L}{}^{\frac{3}{2}}}{C_{D}}$, $t_{FA}=\sqrt{\frac{\rho s}{2G^{3}}}\times \frac{C_{L}{}^{\frac{3}{2}}}{C_{D}}$.

The endurance time can be expressed as follows:

(9) $$t=Q\cdot t_{FA}$$

The meaning of each variable:
*t*—endurance time, s;*Q*—battery discharge energy, J;*G_FA_*—mass coefficient, s/J;*C_FA_*—aerodynamic coefficient;*t_FA_*—time coefficient, s/J;*ρ*—air density,1.185kg/m^3^;*s*—effective projected area, m^2^;*G*—UAV weight, N;*C_D_*—drag coefficient;*C_L_*—lift coefficient.

#### 2.3.2. Feature Factor Extraction Method

According to Equation (8), the battery power *Q* and time coefficient determine the endurance time. During the design of the UAV, the take-off weight and battery power were generally fixed. Therefore, the endurance time was mainly related to the projection area *s*, and the lift coefficient and drag coefficient were related. An UAV typically employs a tapered wing, and the projection area is primarily associated with the root chord length, wingtip chord length, and wingspan length.

**Figure 4:**
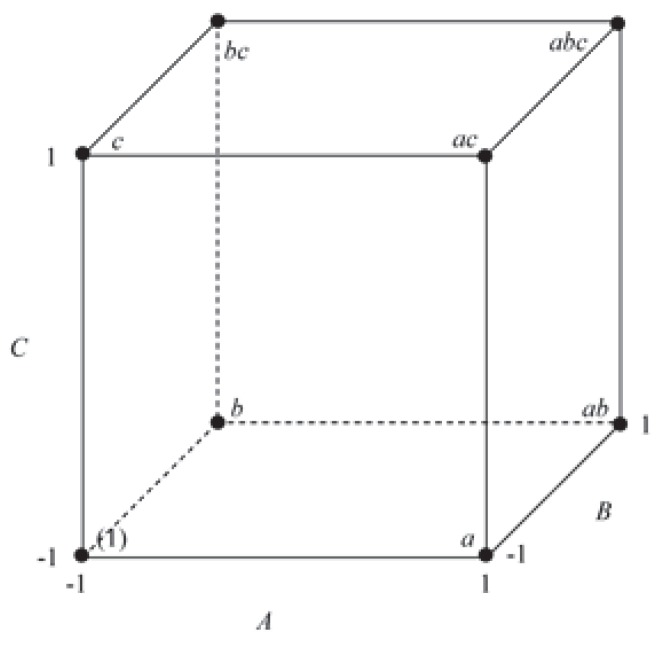
2^3^ sample points.

The two levels of the factor in the 2^k^ design are usually represented as –1 (for the first level) and 1 (for the second level). Note that this representation is reversed from the coding used in general full factorial designs for the indicator variables that represent two level factors in analysis of variance (ANOVA) models [46]. For ANOVA models, the first level of the factor is represented using a value of 1 for the indicator variable, while the second level is represented using a value of –1. When k = 3, the design can be represented geometrically using a cube with the eight treatment combinations lying at the eight corners, as shown in Figure 4.

A two-level factorial design is a type of multi-factor cross-group experimental design [47]. In 1993, Moore and Dalva applied a 2^k^ test for the first time to determine the influence of temperature and groundwater level on the CO_2_ and CH_4_ of soil. Not only were the effects of the two variables on gas emissions investigated but also the effects of their interaction on emissions [48]. Fogue et al. used a statistical analysis based on the 2^k^ factorial methodology to determine the most representative factors that affect traffic safety applications under real roadmaps [49]. The 2^k^ factorial design can test not only the differences between the levels of each factors but also check the interactions between elements [50,51,52].

In this study, a 2^k^ factorial design was used to conduct a full combination test for the maximum and minimum values of the nine structural parameters to obtain 512 sets of experimental data. A variance analysis was carried out for the main effect and second-order interaction effect of each factor to determine their impacts on the endurance time coefficient.

In the half normal plot, the effects with an absolute value exceeding the margin of error (ME) are labeled as significant. The Minitab software was used to calculate the ME. The margin of error is expressed as follows:

(10) ME=t×PSE

where *t* is the (1–α/2) quantile of a *t*-distribution. Lenth’s pseudo standard error (PSE) is based on the concept of sparse effects, which assumes that the variation in the smallest effects is caused by a random error. The PSE was calculated in the Minitab software as follows:

1. To calculate the absolute value of the effects;
2. To calculate Δ, which is 1.5× the median of the effects in step 1;
3. To calculate the median of effects that are less than 2.5 × Δ;
4. To calculate the PSE, which is 1.5× the median calculated in step 3.

#### 2.3.3. Endurance Time Response Surface

**Figure 5:**
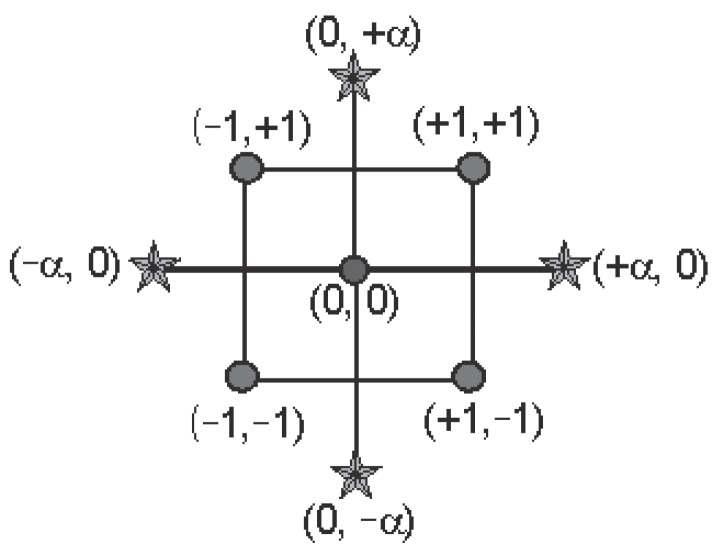
Circumscribed central composite design (CCD) sample points.

Central composite design is a five-level fractional factorial design that is suitable for calibrating the quadratic response model [53]. There are three types of central composite designs (CCDs) that are commonly used in experiment designs: circumscribed, inscribed, and face-centered CCDs. The five-level coded values of each factor are represented by (−a, −1, 0, +1, +a), where (−1, +1) corresponds to the physical lower and upper limit of the explored factor space. It is obvious that (−a, +a) establishes new “extreme” physical lower and upper limits for all factors. Figure 5 is a geometrical representation of a circumscribed CCD of two factors:

The central composite design can analyze the importance of factors and the interactive responses between factors. CCD is widely used in chemical reagent ratios and processing technology optimization [54,55]. In 2006, Idris et al. conducted a central composite design study and built a response surface methodology to identify the essential interfacial reaction factors that influence membrane performance [56]. In the CCD test, each variable has five levels that can be used to fit the response surface and optimize the relevant structural parameters. In 2016, Wei et al. combined the response surface methodology (RSM) and CCD to optimize the structural parameters of a multi-objective analysis and achieved dynamic performance improvement of the cross-spring compliant microdisplacement magnifying mechanism [57].

The four essential influence factors for the time coefficient are root chord, wingtip chord, wingspan, and sweep angle as discussed in Section 3.1. The CCD was used to design the four structural parameters and obtain 16 cubic points, eight axial points, and one center point, which amounts to a total of 25 combined sample points. The CFD method was used to calculate the time coefficient and air resistance values of the 25 combined sample points, and the genetic aggregation response surface method was used to construct the response surface models of the time coefficient *t_FA_* and air resistance ***D***.

Four types of metamodels (polynomial regression, Kriging regression, support vector regression, and moving least squares) with different settings were used as the initial metamodels to build the response surface model. To increase the chance of obtaining the most effective response surface, DesignXplorer generated a population of metamodels with different types and settings [58,59]. The next population was obtained by the cross-over and mutation of previous populations using the genetic algorithm run by DesignXplorer.

The genetic aggregation generated response surface can be expressed as an ensemble using the weighted average of different metamodels, as follows:

(11) $${\hat{y}}_{ens}\left(x\right)=\sum_{i=1}^{N_{M}}w_{i}\cdot {\hat{y}}_{i}\left(x\right)$$

where ${\hat{y}}_{ens}$ is the prediction of the ensemble, ${\hat{y}}_{i}$ is the prediction of the *i-*th response surface, *N_M_* is the number of metamodels used, and $w_{i}$ is the weight factor of the *i-*th response surface.

The weight factors satisfy the following conditions:

(12) $$\sum_{i=1}^{N_{M}}w_{i}=1\;\mathrm{and}\;w_{i}\geq 0,1\leq i\leq N_{M}.$$

### 2.4. Multi-Objective Genetic Algorithm (MOGA)

The tailsitter UAV collects remote sensing images of the target area in the fixed-wing cruising mode. During the design process, it should be ensured that the drone can achieve a long endurance time. During the attitude conversion process, the pitch angle gradually increases from 0° to 90°. To ensure the stability of the attitude conversion process, the air resistance of the drone should be as small as possible:

(13) $$\begin{array}{l} \max t\left(x\right)=\left[t_{1}\left(x\right),t_{2}\left(x\right),\cdots ,t_{n}\left(x\right),\right]\\ \min\Delta D\left(x\right)=\left[\Delta D_{1}\left(x\right),\Delta D_{2}\left(x\right),\cdots ,\Delta D_{n}\left(x\right),\right] \end{array}$$

$$\begin{array}{l} n=1,2,\cdots N\\ s.t.K_{i}\left(x\right)\geq K_{0}\left(x\right)\\ D_{i}\left(x\right)\leq D_{0}\left(x\right)\\ x=\left[x_{1},x_{2},\cdots ,x_{d},\cdots ,x_{D}\right]\\ x_{d\;min}\leq x_{d}\leq x_{d\;max}\;\;\;d=1,2,3,4\\ i=1,2,\cdots ,m;j=1,2,\cdots ,k \end{array}$$

where ***D*** is the air resistance of the UAV; *x* is a structural parameter; *x_d_* is the structural parameter range; and *m, n, i, j,* and *d* denote the number of invariant states.

The MOGA used in this paper was NSGA-II (non-dominated sorted genetic algorithm-II) [60]. The parameters and operators of the NSGA-II were as follows: the population size was 100, the number of generations was 100, the mutation rate was 0.01, the crossover rate was 0.98, the type of crossover was one point, and the type of mutation was simple mutation.

The response surface and optimized MOGA were built with DesignXplorer, which described the relationship between the design variables and the performance of the product by using design of experiments (DOE), combined with the response surfaces [61]. In the DesingXplorer software, the initial population was constructed by the MOGA module, the data of the constructed population were transmitted to the RSM module to solve the endurance time and air resistance, and the solution result was transmitted to the MOGA module for convergence verification [62]. If the requirements were not met, the population crossover and mutation were carried out by the MOGA for a new iterative calculation, which was iterated until finding the optimal individual solution with the longest endurance time and least air resistance, which are the optimal structural parameters.

### 2.5. CFD Model Validation

#### 2.5.1. Test Materials and Equipment

**Figure 6:**
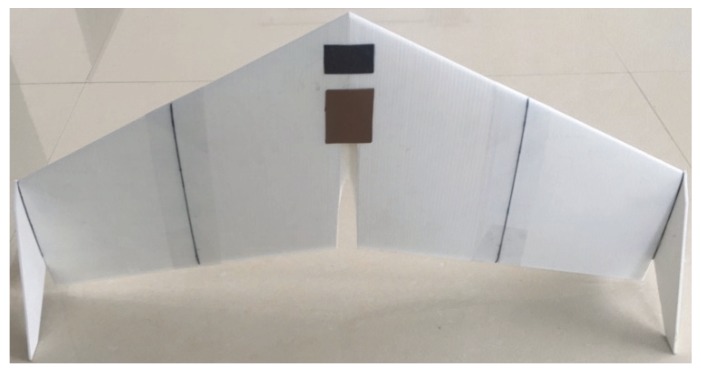
Wind tunnel experimental prototype.

A wind tunnel test was carried out to verify the CFD numerical simulation results. A sample point (wingspan 1200 mm, sweep angle 28°, root chord length 500 mm, and wingtip chord length 280 mm) was randomly selected from 25 CCD combination samples. The aerodynamic coefficients *C_FA_* and ***D*** of the prototype were measured under different wind speeds. Additionally, 3D printing technology was used to process the sample (as shown in Figure 6); the wings of the sample were scaled to 600 mm according to the similar criteria used in wind tunnel tests.

#### 2.5.2. Test Conditions and Scheme

**Figure 7:**
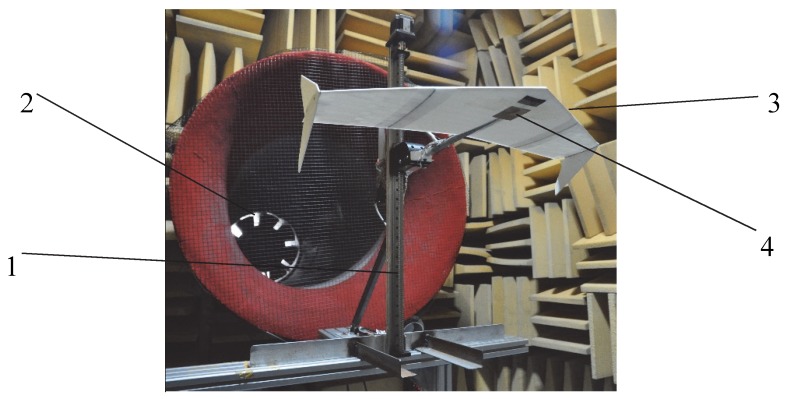
Wind tunnel experiment system. (1) Aircraft support; (2) fan; (3) test UAV sample; (4) six-component strain balance.

The wings and winglets were produced by 3D printing technology and assembled into a UAV sample. The grooves and gaps of the wings were skinned to ensure the surface smoothness. The wind tunnel test system includes aircraft support, six-component strain balance, fan, frequency converter, and data analysis system, as shown in Figure 7. A six-component strain balance was embedded into the UAV body to reduce the interference of airflow to the balance, and the UAV body was fixed with an extension strut to minimize the intervention of the support to the tail airflow. During the test, the UAV was kept at the center of the wind field (1.4 m above the ground) to obtain the best wind field data, and the inverter was used to control the fan speed. After the wind speed was stable, the lift coefficient and drag coefficient were determined by the six-component strain balance. The data analysis system analyzed 1000 data sets and finally obtained the aerodynamic coefficients and air resistance of the drone.

The wind tunnel test was conducted at the Chinese National Key Laboratory of Science and Technology on Aerodynamic Design and Research. During testing, the pitch angle of the UAV was set at 8°, and the wind speed was stabilized between 12 and 20 m/s using a frequency converter at 2 m/s intervals. The lift–drag ratio and air resistance values of the UAV sample were determined at different wind speeds and the simulated values of the CFD was compared with the measured values in the wind tunnel test to verify the accuracy of CFD simulation methods. 

#### 2.5.3. Numerical Simulation Results Verified

**Figure 8:**
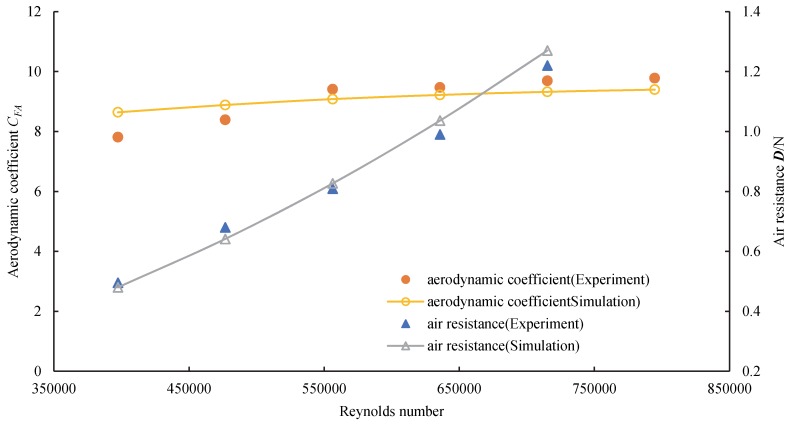
Wind tunnel test results.

Change in the UAV aerodynamic operating conditions is essentially a change in the Reynolds number. According to the Reynolds number similarity theory and geometric similarity, the same Reynolds number produces similar aerodynamic characteristics. The scatter diagrams for the aerodynamic coefficient *C_FA_* and ***D*** of the UAV under different Reynolds numbers were drawn and compared with the wind tunnel data as shown in Figure 8.

The average error of the aerodynamic coefficient *C_FA_* was less than 5%, while the air resistance ***D*** less than 6%. Since the UAV model was constructed using 3D printing, the surface roughness affected the flow field distribution close to the wings. The model’s air resistance was small, and the model’s jitter, signal interference, and other factors in the test process affected the accuracy of the air resistance measurements. The errors in the test results were less than 6% within the allowable range, which indicates that the CFD numerical simulation method is reliable and can simulate the outflow field of tailsitter UAVs.

## 3. Results and Discussion

### 3.1. Feature Factor Extraction Results

**Figure 9:**
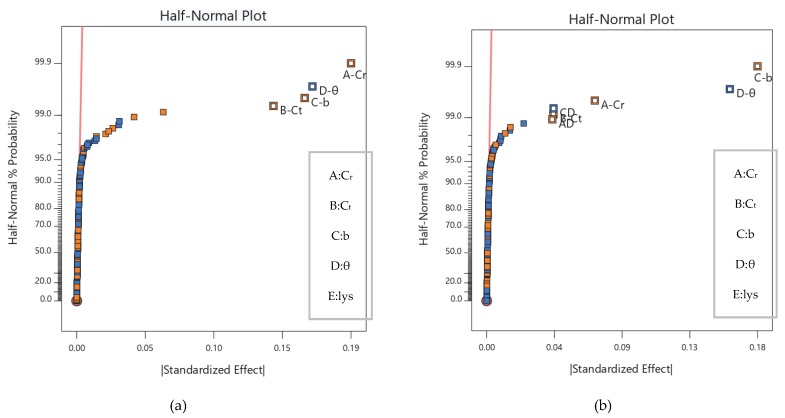
Half-normal distribution of the structural parameters. (**a**) Air resistance half-normal graph; (**b**) time coefficient half-normal graph.

Figure 9 shows the half-normal distribution of the nine structural parameters with the time coefficient and air resistance.

The half-normal distribution chart shows that the specific factors of the time coefficient and air resistance are the root chord, wingtip chord, wingspan, and sweep angle. In the local sensitivity of air resistance, the root chord, wingtip chord, and wingspan were positively affected, while the sweep angle was negatively affected, and the sensitivity was *Cr* > *b* > *C_t_* > *Λ_w_*. In time coefficient terms, the wing root chord length and wingspan length were positively affected, while the sweep angle and wing chord length were negatively affected, and the sensitivity was *b* > *Λ_w_* > *C_r_* > *C_t_*.

The mathematical expression of air resistance and time coefficient is

$$100\cdot D=-33.324+0.097\cdot C_{r}+0.093\cdot C_{t}+0.054\cdot b-0.278\cdot \theta$$

$$1000\cdot t_{FA}=-236+0.139\cdot C_{r}+0.29\cdot C_{t}+0.733\cdot b-0.410\cdot \theta +0.007\cdot C_{r}\cdot \theta -0.005\cdot b\cdot \theta$$

It can be seen from the mathematical expressions and Figure 9 that there are no interactions between the four influence factors of air resistance, however, there is an interaction between the wingspan and sweep angle, as well as between the root chord length and sweep angle for time coefficient. The aerodynamic coefficient of the drone is mainly determined by the shape and the incoming angle. Moreover, the two factors interact in the distribution of the fuselage flow field. The sweep angle is the main factor affecting the incoming angle, and the wing root and wingspan are the main factors of the shape. The interactions between the wingspan, sweep angle, and root chord length should be considered when calculating endurance time.

### 3.2. Effects of the Structural Parameters on the Time Coefficient and Air Resistance

#### 3.2.1. Effects of the Structural Parameters on the Time Coefficient

**Figure 10:**
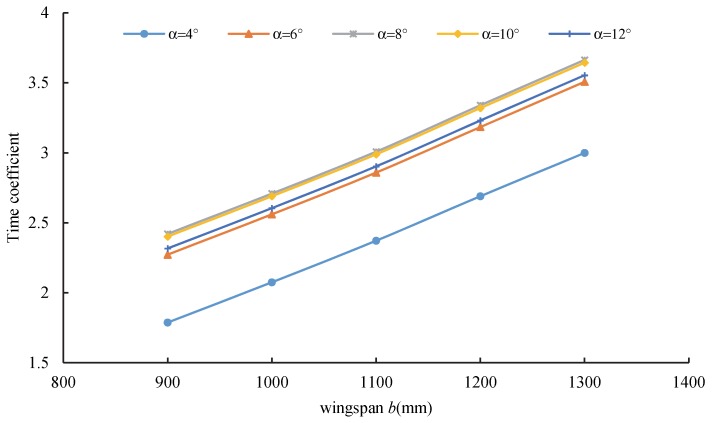
Relationship between the wingspan and time coefficient.

Figure 10 shows the relationship between the wingspan and the time coefficient. Under the specified attack angle, the time coefficient increased with an increase of wingspan *b*. The endurance time was determined by the mass coefficient and aerodynamic coefficient. The mass coefficient was determined by the projected area, which was positively correlated with wingspan *b.* The aerodynamic coefficient was related to the lift–drag ratio, which was determined by the aspect ratio. Therefore, the aspect ratio had a linear relationship with the wingspan length.

**Figure 11:**
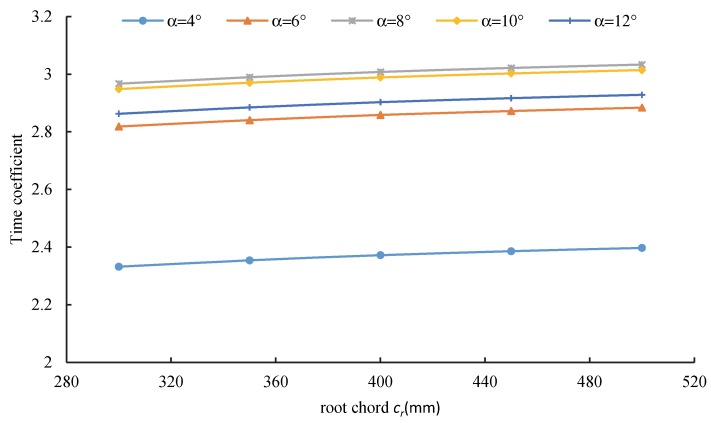
Relationship between the wing root chord length and time coefficient.

Figure 11 shows the variation of the time coefficient with the wing root chord length under different attack angles. At the same angle of attack, the time coefficient increased with the wing root chord length. When the attack angle was 8°, the time coefficient with a wing root chord length of 500 mm was 3% higher than that with a wing root chord length of 5 mm. 

**Figure 12:**
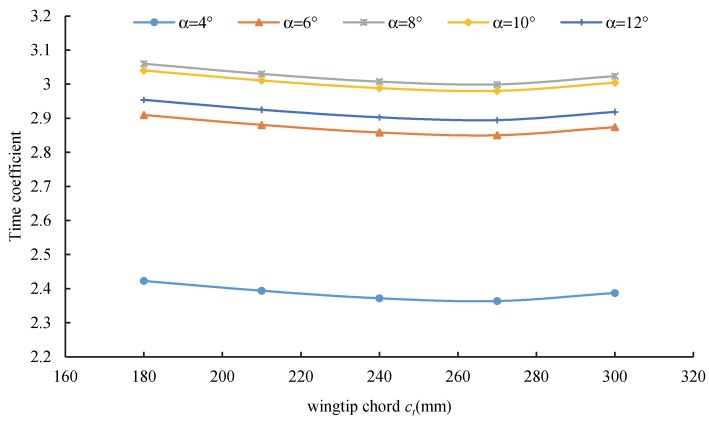
Relationship between the wingtip chord length *c_t_* and time coefficient.

Figure 12 shows the variation of the time coefficient with the wingtip chord length under different attack angles. The results illustrate that the time coefficient first decreased and then increased as the wingtip chord length increased. Under the specified wingtip chord length, there existed a minimum time coefficient value. Moreover, the time coefficients with wingtip chord lengths of 180 mm and 300 mm were 2.6% and 0.8% higher than those with a wingtip chord length of 270 mm, respectively. As the size of the wingtip increased, the turbulence of the wing tip gradually weakened, while the projected area of the wing increased. Accordingly, the endurance time first exhibited a decreasing, and then an increasing, trend with an increase of the wingtip.

**Figure 13:**
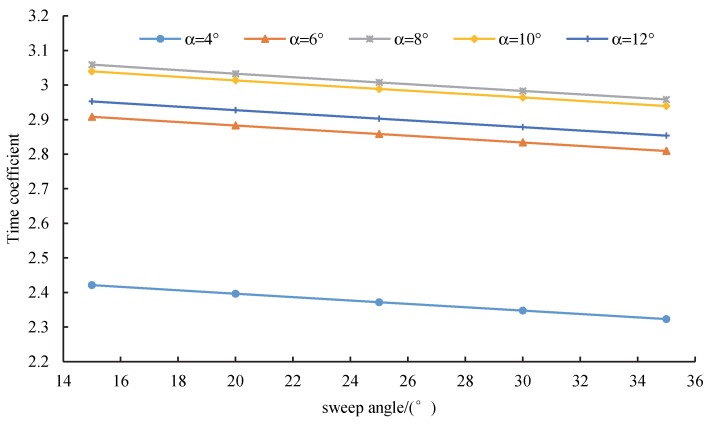
Relationship between the sweep angle and time coefficient.

Figure 13 shows the variation of the time coefficient with the wing sweep angle under different attack angles. When the sweep angle increased from 15° to 35°, the time coefficient of the UAV linearly decreased. The time coefficient with a sweep angle of 35° was 4% lower than that with a sweep angle of 15°. This result can be explained by the aerodynamic coefficient *C_FA_* when the sweep angle changed. As the sweep angle increased, the lift coefficient *C_L_* first increased and then decreased. The lift–drag ratio *K* first decreased and then increased, and the combination of the two exhibited a linear downward trend. Under the specified attack angle, the aerodynamic coefficient *C_FA_* increased with the wing sweep angle *Λ_w_*, and the wing sweep angle had less of an impact on the mass coefficient *G_FA_*. Thus, the time coefficient increased with the wing sweep angle in a specified geometrical structure.

When the angle of attack changed from 4° to 12°, the time coefficient increased in the range of 4–8° and decreased in the range of 10–12°. The endurance time significantly declined with an attack angle of 6°. As the angle of attack increased, the laminar flow effect on the wing surface gradually weakened, the turbulence effect intensified, the aerodynamic coefficient efficiency of the UAV decreased, and the change rate of the endurance time decreased.

#### 3.2.2. Effects of the Configuration Parameters on Air Resistance Performance

**Figure 14:**
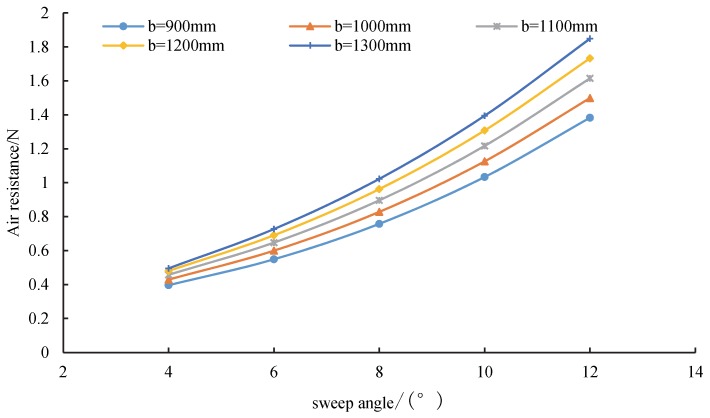
Variation of air resistance under different attack angles.

Figure 14 shows the relationship between the wingspan and air resistance. Under the specified attack angle, the air resistance increased with wingspan *b*. Moreover, the rate of the air resistance growth became faster as the wingspan increased in a specified geometrical structure, which can be explained by the differential pressure between the upper and lower wing surfaces. Owing to the effect of the air pressure difference on the wingtip, their induced resistance was present from the bottom–up. As the wingspan increased, the induced air resistance increased, and thus the air resistance increased.

**Figure 15:**
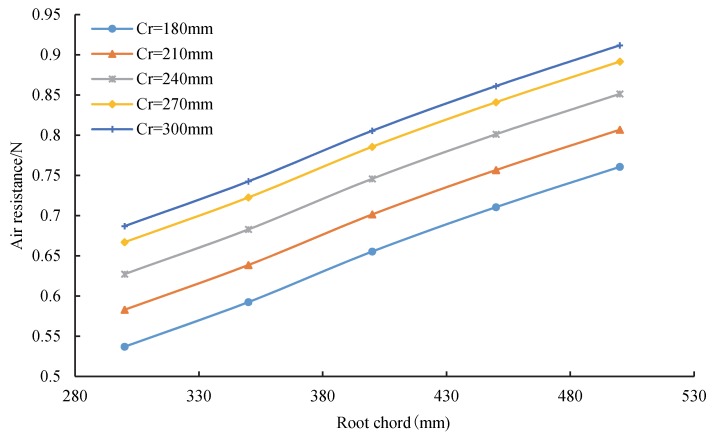
Relationship between the air resistance and wing root chord for different wingtip chord.

The air resistance variation with the root chord length under different wingtip chord lengths is shown in Figure 15. As can be seen, the air resistance increased with the root chord length under the specified length of the wingtip chord. When the wingtip chord length changed from 180 to 240 mm, the air resistance increased under the specified length of the wing root chord length. Additionally, the growth rate of the air resistance increased at 180–240 mm and gradually decreased at 270–300 mm. 

This result can be explained by the wing root effect on the swept-wing. When the air flowed through the swept-wing, the streamline was slightly skewed toward the wingtip, which resulted in an s-shaped streamline distribution. On the upper surface of the wing root, the streamline exhibited an expanding trend at the front region, and the streamline exhibited a shrinking trend in the back area. The offset of the streamline increased with the wing root chord length and wingtip chord length, which increased the air resistance on the wing. The root–tip ratio decreased with an increase of the wingtip chord length, which reduced the induced resistance of the wingtip. The air resistance gradient decreased as the induced resistance decreased. In short, the air resistance increased with the wing root chord length and wingtip chord length.

### 3.3. Multi-Objective Optimization

**Figure 16:**
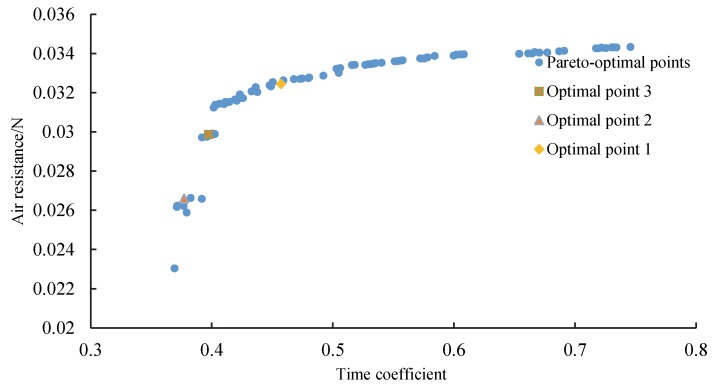
Multi-objective genetic algorithm (MOGA) optimization.

Multi-objective optimization, including the maximum *t_FA_* and minimum ***D***, was applied to obtain a set of optimal solutions (as shown in Figure 16). The initial population was constructed using the screening method, and the time coefficient and air resistance were calculated using the response surface model. Table 3 presents the three optimized configurations. Compared with the original prototype, the time coefficient *t_FA_* of optimal configuration 1 was enhanced by 19.5%, while the air resistance ***D*** was reduced by 34.78%. For all three optimal configurations, the time coefficient *t_FA_* increased by an average of 10.5%, while the air resistance ***D*** was reduced by an average of 40.58%. This study selected optimal configuration 1 as the solution with the best compromise. The parameters of the final tailsitter UAV structure are a wing root chord length of 315 mm, a wingtip chord length of 182 mm, a wingspan length of 1198 mm, and sweep angle of 16°.

In this study, three optimal configurations were simulated and verified. The CFD simulation value was used as the actual value for comparison with the response surface prediction value, as presented in Table 3. The relative error of ***D*** and *t_FA_* increased with the sweep angle. For all three verification points, the relative error of the time coefficient *t_FA_* was less than 3%, while the relative error of the air resistance ***D*** was less than 5%. 

Based on previous research and increasing demand for agricultural remote sensing, a VTOL UAV was designed with vertical take-off and landing characteristics, as well as dynamic cruise characteristics. These characteristics solve the low efficiency and short-range of failures for multi-rotor UAVs, the lengthy fixed-wing UAV take-off preparation, auxiliary equipment requirements, and various landing problems. Ang et al. designed a foldable tailsitter U-lion in 2014 [12]. The wing of this U-lion could be retracted through a link mechanism, which improved the maneuverability and portability of the drone. Aksugur and Inalhan designed a ducted tailsitter drone and adjusted the attitude in the vertical take-off and landing state by using a ducted steering gear [19]. In contrast, our tailsitter UAV used a dual-wing symmetrical layout, and there was no additional auxiliary mechanism for attitude adjustment. This UAV has the characteristics of a compact structure, light weight, and smooth operation.

The predecessors of this system mainly used CFD calculations to compare the lift–drag ratio and air resistance of each sample in the discrete sample points to find the optimal sample point. Cetinsoy et al. used CFD simulations to calculate the winglet turbulence under three airfoils and two different angles of attack. The optimal winglet size was determined by the wingtip vortex and lift [16]. Rao et al. used CFD simulations to analyze the UAV’s drag coefficient and the lift–drag ratio at ten wind speeds and nine angles and determined the aerodynamic characteristics of the UAV in transition mode [14]. In this study, a calculation model of endurance time was established, and a response surface model of endurance time and air resistance was constructed. A multi-objective genetic algorithm was used to determine the optimal structural parameters. Unlike previous studies, this study adopts battery life as a comprehensive performance index to evaluate the versatility of tailsitter UAVs. The response surface model has sufficient continuity to achieve the optimization of global structural parameters. Compared with the original prototype, the final solution structure with a good compromise was determined by using the multi-objective genetic algorithm to improve the time coefficient by 19.5% and reduce the air resistance by 34.78%. These results show that a multi-objective genetic algorithm based on the genetic aggregation response surface can be used to optimize tailsitter drones.

## 4. Conclusions

This study designed an agricultural VTOL tailsitter UAV with symmetrical winglets and wings. The time coefficient and resistance were calculated under different structural parameter combinations using the CFD numerical simulation method. Additionally, a response surface model of the time coefficient and air resistance with genetic aggregation was developed, and the structural parameters were optimized using a multi-objective genetic algorithm.

(1) The root chord, wingtip chord, wingspan, and sweep angle were the specific factors that influenced the time coefficient and air resistance. In terms of air resistance, the root chord, wingtip chord, and wingspan were positively affected, while the sweep angle was negatively affected, and the sensitivity was Cr > *b* > *C_t_* > *Λ_w_*. In time coefficient terms, the wing root chord length and wingspan length were positively affected, while the sweep angle and wing chord length were negatively affected, and the sensitivity was *b* > *Λ_w_* > *C_r_* > *C_t_*.

(2) The CFD simulation was verified by conducting a wind tunnel test, and the results revealed that the aerodynamic coefficient error was less than 5%, while the resistance error was less than 6%. This result indicates that the CFD numerical simulation method can be used to simulate the external flow field of a tailsitter UAV.

(3) The time coefficient increased with the wing root chord length and wingspan, decreased with an increase of the sweep angle, and first decreased and then increased as the wingtip chord length increased. The air resistance increased as the wing root chord length, wingtip chord length, and wingspan increased, and first decreased and then increased with an increase of the sweep angle. 

(4) The solution parameters for the tailsitter UAV with the best compromise were a wing root chord length of 315 mm, a wingtip chord length of 182 mm, a wingspan length of 1198 mm, and a sweep angle of 16°. Compared with the original shape, the time coefficient *t_FA_* was enhanced by 19.5% while the air resistance ***D*** declined by 34.78%. 

## Figures and Tables

**Table 1:** Electric driven fixed-wing UAV parameters.

Product Name	Take-off Mass/kg	Cruise Speed/m·s^−1^	Wingspan/mm
senseFly	0.5	12	780
senseFly eBee	0.6	11	960
Parrot Disco-Pro	0.7	12	1150
skywalker X5	0.9	12	1200

**Table 2:** Parameter range of the tailsitter UAV.

*c_r_*/mm	*c_t_/*mm	*b*/mm	*Λ_w_*/(°)	*l_ys_*/mm	*l_v_*/mm	*Λ_v_*/(°)	*h*/mm	*l_jc_*/mm
240~500	150~300	800~1200	0~60	100~130	30~60	30~60	5~25	70~150

**Table 3:** The optimal structural parameters.

Parameters	Original Prototype	Optimal Point 1	Validate 1	Optimal Point 2	Validate 2	Optimal Point 3	Validate 3
root chord/mm	400	314.7		316.6		308.1	
wingtip chord/mm	240	181.5		184.3		186.5	
wingspan/mm	1000	1197.8		1147.2		1037.9	
sweep angle/(°)	25	15.9		32.7		31.1	
endurance time*100	2.72	3.25	3.19	2.99	2.91	2.66	2.60
Predicted and relative error/%		19.5	1.88	9.93	2.75	2.21	2.34
air resistance/N	0.69	0.45	0.46	0.40	0.42	0.38	0.39
Predicted and relative error/%		34.78	2.17	42.02	4.76	44.93	2.56
